# Two-Layered Multi-Factor Authentication Using Decentralized Blockchain in an IoT Environment

**DOI:** 10.3390/s24113575

**Published:** 2024-06-01

**Authors:** Saeed Bamashmos, Naveen Chilamkurti, Ahmad Salehi Shahraki

**Affiliations:** Department of Computer Science and Information Technology, La Trobe University, Bundoora, Melbourne 3086, Australia; n.chilamkurti@latrobe.edu.au (N.C.); a.salehishahraki@latrobe.edu.au (A.S.S.)

**Keywords:** biometric, blockchain, digital signatures, IoT, multi-factor authentication, PUF

## Abstract

Internet of Things (IoT) technology is evolving over the peak of smart infrastructure with the participation of IoT devices in a wide range of applications. Traditional IoT authentication methods are vulnerable to threats due to wireless data transmission. However, IoT devices are resource- and energy-constrained, so building lightweight security that provides stronger authentication is essential. This paper proposes a novel, two-layered multi-factor authentication (2L-MFA) framework using blockchain to enhance IoT devices and user security. The first level of authentication is for IoT devices, one that considers secret keys, geographical location, and physically unclonable function (PUF). Proof-of-authentication (PoAh) and elliptic curve Diffie–Hellman are followed for lightweight and low latency support. Second-level authentication for IoT users, which are sub-categorized into four levels, each defined by specific factors such as identity, password, and biometrics. The first level involves a matrix-based password; the second level utilizes the elliptic curve digital signature algorithm (ECDSA); and levels 3 and 4 are secured with iris and finger vein, providing comprehensive and robust authentication. We deployed fuzzy logic to validate the authentication and make the system more robust. The 2L-MFA model significantly improves performance, reducing registration, login, and authentication times by up to 25%, 50%, and 25%, respectively, facilitating quicker cloud access post-authentication and enhancing overall efficiency.

## 1. Introduction

Emerging IoT is a wave technology in wireless communication supporting diverse applications using cost-efficient sensors, actuators, and smart devices [1]. Even though communication in an IoT environment is limited by human intervention, providing end-to-end (E2E) data confidentiality and privacy is essential. An IoT device aggregating information from surrounding and IoT users who access the collected data is supposed to require authentication on both sides [2]. A trusted third party (TTP) in IoT is enabled to mitigate the computations at devices. Identity, password, and signature are the two major metrics used for authentication [3]. Hash-function-based encryption and decryption are also associated with authentication; hence, IoT prefers lightweight algorithms. PUF is a special hardware presented in IoT devices to ensure security [4]. PUF can be called a digital fingerprint developed uniquely for a particular device. The major attacking threats, such as man-in-middle attacks (MIMT), insider attacks, impersonation attacks, replay attacks, and others, are overwhelmed by PUF [5]. Silicon PUF is built with a one-way function and generates pairs of challenge–response for individual devices. Based on this challenge–response pair, the PUF is categorized into strong and weak. PUF is a promising security primitive with the potentialities of reliability, unpredictability, and uniqueness.

Cloud IoT is employed to provision remote access to users [6]. Global connectivity is also a major reason for security. The key security requirements and features include data integrity, availability, key agreement, mutual authentication, anonymity, and forward secrecy. Smart sensing with IoT devices is highly resource-constrained. Hence, it demands lightweight authentication, which can be provided as signature-based, radio frequency identification (RFID), pseudonym identity, and factors-based authentication [7,8,9]. Smart cards are popularly used for authentication, enabling them to store security attributes used during authentication. MFA (multi-factor authentication) considers more than one significant attribute for identifying individual users [10]. The primary factors for MFA are passwords, biometrics, and smart devices. Legitimate users are predicted from two-factor authentication whose constraints are PUF with location information [11]. Location is predicted from two signal characteristics: the signal strength indicator (RSSI), and the link quality indicator (LQI). The time-based one-time password (TOTP) is computed to exchange initial parameters as pseudonyms and emergencies. The reference node assists in determining location, which is used as a significant factor for authentication. Authentication in IoT also allows the participation of different cryptography techniques (citation). At this moment, PUF is the best solution for security in an IoT environment.

Biometrics is one of the important factors that are presented for authentication. The fingerprint is the most used biometric for identifying individuals [12]. The fingerprint is encrypted and stored; further, during authentication, it is retrieved back for verification. A lightweight biometric-based remote user authentication scheme is proposed by handling hash and XOR operations [13]. Recently, biometric recognition has been supported over diverse biometric entities such as fingerprint, iris, palm, and many more. They are imposed to have distinct features, so the biometric authentication is ensured to be accurate. To minimize computations, one-way hashes and perceptual hash functions are produced in simple operations. An iris-based authentication scheme is proposed along the hybrid cryptography of the blowfish and RSA algorithms [14]. The combination of two encryption algorithms ensures higher security in the system, and the use of iris peculiarly identifies legitimate users. Therefore, in this paper, an efficient MFA in IoT infrastructure based on multiple factors is designed with decentralized blockchain technology.

Specifically, the major contributions of this paper are depicted as follows.

- The 2L-MFA blockchain technology protects IoT devices and cloud-stored data. Even with authentication, IoT data are secured using a lightweight elliptic curve Diffie–Hellman. Data management begins with IoT devices and user registration in the cloud. Logging onto the cloud authenticates users. IoT devices uploading data to the cloud are approved. Initial tiers authenticate IoT devices’ identification, secret key, location, and SRAM PUF. Blockchain verifies entities in the system PoAh authenticates lightly.
- Four individual-factor authentication sublevels are available for second-tier IoT user authentication. Level 1 verifies identity and password via matrix-based password enrolment. Biometrics is used at levels 3 and 4, whereas signatures are used at level 2. Moreover, XOR operations create a binary key from iris biometric data at level 3. Level 4 finger vein and biometric binary key authentication occur. Pixel difference equals finger vein.
- IoT user authentication is checked using fuzzy logic. If suspicious, the user’s next authentication is strengthened in level 2 on challenge–response. The proposed 2L-MFA in the cloud with blockchain is assessed for registration, login, authentication, and success rates.

The rest of this paper is structured into the following sections: Section 2 addresses the previous research works in IoT authentication and their limitations; Section 3 details the peculiar problem existing in MFA over IoT; Section 4 is composed of all solutions for IoT security; Section 5 presents experimental evaluation for identifying the efficiencies of proposed work; and Section 6 concludes the proposed authentication beside their future directions.

## 2. Related Works

The paper [15] cryptanalyzes their method and finds it vulnerable to stolen verifiers and smart card assaults. To fix their strategy, they released an improved one. Its formal security study, which employs the random oracle model, and informal security analysis show that the method is secure against various known attacks. Its formal security verification uses ProVerif. ECDLP, which employs hash and XOR functions for authentication in cloud-based Internet of Things devices, is a novel and effective method based on the elliptic curve feature. The recommended protocol is more attacker- and flaw-resistant than existing methods. XOR and one-way hash provide effective cost reduction for processing. AVISPA and BAN logic were used to analyze the proposed protocol [16].

The study [17] proposes a three-factor authentication method for the Internet of Things that is both lightweight and resilient. It is based on the linear feedback shift register (LFSR). Furthermore, the majority function is used to improve end-to-end device security and do away with the linear structure of LFSRs. The suggested technique can withstand replay, impersonation, eavesdropping, and man-in-the-middle attacks as it offers anonymous property and forward secrecy, according to the informal security study. The study [18] examines the frequent flaws and difficulties in creating a user authentication method for cloud-assisted Internet of Things devices using a standard technique presented at IEEE TDSC 2020. They also provide a fresh approach to safe user authentication, requiring less gateway processing power. The suggested system has several desirable attributes, including forward secrecy and multi-factor security, to enable safe connection between distant users and IoT devices. The random-oracle model, heuristic analysis, the ProVerif tool, and BAN logic are used to demonstrate the security of this method in the interim.

The study [19] introduces CMAF-IIoT, a chaotic map and resource-efficient AE scheme (ASCON)-based authentication framework for IIoT. SMDs and users may communicate with confidence thanks to CMAF-IIoT. The framework starts with user-performed local authentication on their smart devices, followed by mutual authentication with the gateway and the formation of a session key with the SMD. Users may safely access real-time data from SMDs placed across the IIoT ecosystem using the session key. They provide a safe and private authentication method that improves MEC environment security [20]. The suggested method eliminates the requirement for a reliable third party by establishing a secure session key between the user and the MEC server. The physical-features-based key management and authentication approach for wireless networks and Internet of Things networks is proposed in this study [21]. The public key is generated based on the best qualities extracted from network traffic data in this process. The behaviour and data trust values combine to provide the total trust value of each device, which is considered a unique attribute and used to create the private key.

The work [22] used continuous microphone monitoring to verify breathing patterns for implicit IoT authentication. BTL Auth, an on-phone authentication software powered by Tensor Flow Lite and driven by breathing data, uses a neural network model to validate the target user and an audio processing pipeline to filter and compute features. The authors offer energy-efficient RF fingerprinting for physical layers in the study [23], which is specially tailored for IoT nodes with limited resources. They suggest making a little change to the radio front by adding a digital physically unclonable function (PUF) to improve the identification performance over earlier experiments using off-the-shelf radios. The PUF enhances the uniqueness and identification space of the transmitter (TX) beyond just depending on transistor intrinsic process changes by controlling the transmitter’s (TX) spectrum regrowth as the RF fingerprint (RFF). The 45 nm CMOS SOI technology from GlobalFoundries incorporates 2.4 GHz physical layer identification as a proof of concept. The article [24] presents a paradigm for cloud-edge device collaborative device authentication that utilizes physical unclonable function (PUF) technology. Considering the many kinds of devices, the suggestion is to use the parts of the device to construct a PUF, model the PUF using machine learning techniques, and store the PUF model that results for the device in the edge server node. The authors also provide device authentication strategies for cross-domain and intra-domain authentication, respectively, to satisfy the demand for effective and safe mutual authentication. The main technologies used in the suggested authentication architecture and additional PUF technology application possibilities in distributed cloud systems are finally covered.

In the research of [25], the authors provide a remote user authentication technique and symmetric key exchange algorithm that is efficient, safe, and lightweight. The study proposal offers a defence against known assaults with an effective privacy-preserving technique for distant users. The work [26] presents a novel blockchain-based information-sharing system for zero-trust environments that ensures participant stimulation without compromising fairness, data privacy without compromising data trustworthiness, and anonymity while preserving entity authentication. This innovative approach can stop unauthenticated individuals from distributing false information by using consensus methods, smart contracts, and efficient voting to filter out fake content. Furthermore, the authors assessed the suggested solution’s effectiveness on an Ethereum-based blockchain platform to show its value and establish its security within the context of universal composability. The study [27] suggests a unique way to improve security, data integrity, user privacy, system scalability, and device interoperability in Internet of Things services via blockchain technology. To achieve this, smart contracts are given data sharing, authentication, and access control for Internet of Things devices. By building and implementing a smart contract over the Polygon blockchain network in a mock real-world Internet of Things scenario, the suggested methodology was validated. The study [28] suggests a quick reaction time and high security for a lightweight, blockchain-based, Fog-enabled remote patient monitoring system. The suggested lightweight blockchain architecture is safe from assaults and suits the resource-constrained IoT devices effectively, according to simulation findings and security research.

The main goal of this work was to resolve the disputes between users and IoT devices. Illegitimate users can predict conventional signature algorithms in blockchain, and hence the level of privacy is backward. A decentralized blockchain framework was employed with PUF for legitimate user identification and authentication [29,30]. Blocks in the intended blockchain included a header with the block number, Merkle hash tree, and timestamp. Block transactions have signatures and PUFs. Regarding ECDSA-generated signatures, ECDSA’s old method requires more calculations and resources. For blockchain IoT infrastructure security, attribute-based access control was created [31]. This system model addressed IoT devices and attribute authority. Blockchains handle distributed attributes as attribute authorities. IoT devices receive a public and private key during registration following identification verification. The attribute authority generated keys for all registered users. A data-sharing session is exchanged between authorized devices. Here, the properties are merely numbers and words, but storage occupancy is lower; therefore, this is not enough to forecast a legal device.

**Figure 1:**
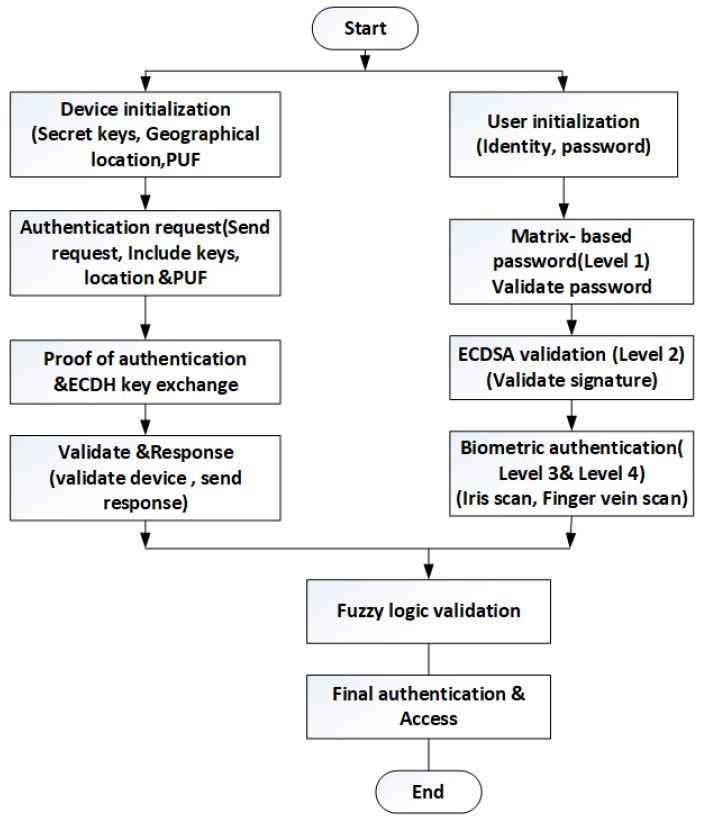
Flow chart for the proposed work.

Cryptography and hashing-based authentication were also addressed in previous research. Elliptic curve cryptography (ECC) was the most widely used technique to ensure security [32,33]. The IoT device chooses a random number to the ECC point. During authentication, this ECC point was verified, and if the point of the device was not true, then the request was rejected, or else authentication would be completed successfully. After authentication is completed, a session key is established to transmit messages between devices. In other work, a customizable BLAKE2b hashing algorithm was integrated with modified ECDSA. The nodes deployed in the environment were responsible for generating key pairs and a random number based on the elliptic curve. The signature was received from the user and verified by the base station in the authentication phase. Here, a signature is a factor for authenticating the originality of the device. Security in an IoT environment with blockchain and PUF would be a better solution, but still, the security factors depend on the achievement of authentication. Figure 1 represents the proposed work flowchart.

## 3. Problem Statement

In the rapidly expanding Internet of Things (IoT), the security of devices and user authentication has become paramount. However, many IoT systems currently rely on single-factor authentication (SFA) mechanisms, which may not provide sufficient security in the face of sophisticated cyber threats.

The reliance on SFA in IoT leaves both devices and users vulnerable to multiple kinds of attacks:

- Authentication challenge: A specific issue with key updates arises when physical unclonable functions (PUFs) are used in security systems. This problem arises because PUF-derived keys are difficult to update compared to traditional cryptography keys. This intrinsic rigidity is a serious security risk, especially when it comes to the possibility of crucial exposure to potential threads.

These vulnerabilities can have far-reaching consequences:

- Insecure aggregation process: The aggregation process’s security and robustness are critical. Aggregation using trustworthy execution environments based on SGX is a step in the right direction. Nonetheless, ensuring the aggregation process’s total security and privacy continues to be a difficult and varied task.
- Insecure aggregating process: The security and resilience of the aggregating process are crucial. A positive step is an aggregation using reliable SGX-based execution environments. However, guaranteeing complete security and privacy of the aggregation process remains a challenging and multifaceted undertaking.

Therefore, this research aimed to develop a novel two-layered multi-factor authentication (2L-MFA) accompanied by a decentralized blockchain in an IoT environment. The first-level-authentication is for IoT devices, which consider secret keys, geographical location, and physically unclonable function (PUF) [34]. Second-level authentication is for IoT users, which are sub-categorized into four levels, each defined by specific factors such as identity, password, and biometric (including iris and finger vein).

This research aims to create an MFA system that

- offers robust security and employs multiple authentication factors beyond passwords, significantly reducing the risk of unauthorized access.
- Preserves user privacy: Minimises data collection and processing, protects user anonymity, and adheres to strict privacy regulations.
- Accommodates resource constraints: Considers many IoT devices’ limited processing power and storage capabilities.
- Ensures usability and seamless user experience: Offers convenient and user-friendly authentication methods that do not significantly impede legitimate access.

## 4. Proposed System

### 4.1. 2L-MFA System Model

The proposed 2L-MFA in IoT is a decentralized blockchain-associated model that ensures secure authentication. A collection of n IoT devices and N IoT users, as $U_{N}=u_{1},u_{2},u_{3},\ldots \ldots$ in the proposed environment, provide back-to-back authentication using blockchain technology. Blockchain technology is emerging as a solution for provisioning security among participating entities. The composition of chain-structured blocks is modelled as a blockchain which records transactions. The stored records cannot be altered, as the records are chained as blocks. Individual blocks are built with cryptographic hashes of the prior block to authenticate the block. The decentralized blockchain independently authenticates, and hence it does not require the involvement of a third party.

Information from IoT devices will be enrolled into the cloud after authentication is completed. In this work, the factors for authenticating an IoT device and user are independent. IoT device authentication factors are identity, secret key, and its own position. On the other hand, IoT users are authenticated with identity, password, signature, and biometrics. Two biometrics, the iris and finger vein, are consecutively authenticated. The IoT devices are equipped with static random-access memory (SRAM)-based PUF adopted as a security component. The potential benefits of SRAM-PUF include the establishment of a unique silicon fingerprint that can be transformed into a cryptographic key with greater strength [35]. The proposed SRAM-PUF addresses all the challenges in present and future security. SRAM-PUF is guaranteed with security and a lifetime; it is assumed to be active for up to 25 years. Thus, the SRAM-PUF is a good IoT solution that is comprised of resource-constrained devices.

**Figure 2:**
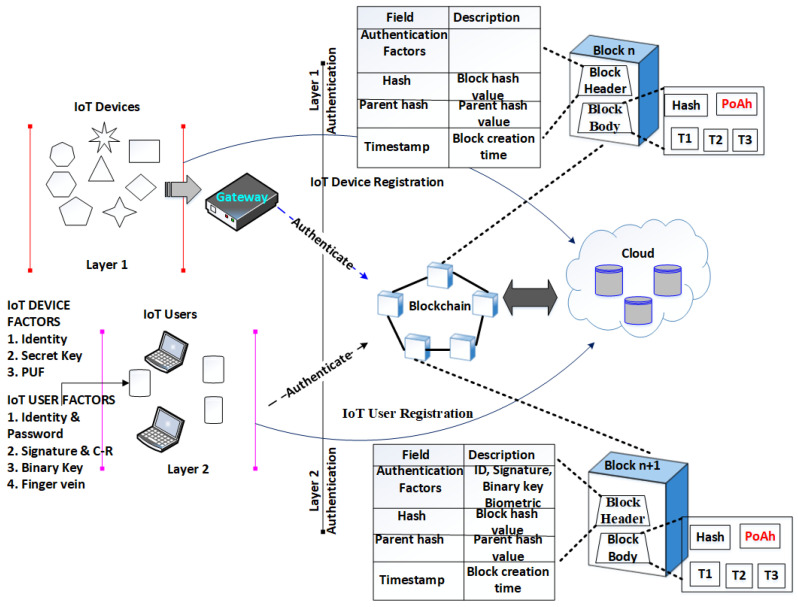
The designed 2L-MFA system.

The proposed two-layered multi-factor authentication (2L-MFA) encompasses two layers: (1) IoT device authentication and (2) IoT user authentication. We present the most significant factors for appropriate authentication by eliminating illegitimate access into the system. Figure 2 depicts the proposed IoT environment.

As discussed above, initial device-level authentication in an IoT ecosystem aims to build trust and security at the device level. This includes basic steps to verify IoT device authorization and protect network and system connections. An essential part of IoT system security is user-level authentication, ensuring that only authorized users can access the system’s resources and data. Authentication on the blockchain with unique factors ensures the allowance of legitimate entities. Blockchain technology strives to offer an accessible, unchangeable ledger to track data origin and preserve data integrity. Blockchain precisely records every transaction and modification IoT devices make as they collaborate to train machine learning models and share updates. Participant authentication of the validity and source of data contributions is made possible by the resulting unchangeable record of data modifications. This audit trail is important in determining data correctness and source credibility in conflicts or abnormalities. For real-time applications, it is very beneficial. Scalability and affordable analytics are made possible by cloud services’ centralized administration and monitoring. To save bandwidth and storage costs while protecting critical data, data are filtered locally and only relevant information is transmitted to the cloud. Table 1 shows the symbolic representations that are used in this paper. The functioning of individual layers is detailed in the forthcoming sections.

### 4.2. First Layer Authentication

Recently, IoT devices have been deployed in many real-world applications and in day-to-day life. This layer focuses entirely on the authentication of IoT devices whose data are submitted to the cloud via blockchain. Each IoT device is presumed to have a unique identity, secret key, and location. The data from these devices are gathered by a secure gateway and delivered to the cloud. SRAM-PUF is a non-cloneable chip that is applied to blockchains for authentication [36]. In the proposed work, each block in the blockchain comprises a block header and block body. The block header contains the authentication factors and other common fields, whereas the block body in the blockchain maintains the transactions. According to the proposed work, the blockchain, as an intermediary, is responsible for authenticating IoT devices. Since IoT devices are resource-constrained, a lightweight elliptic curve Diffie–Hellman method is used for secure data storage. The blockchain authenticates the secret key, PUF, and location. This layer is constructed with three phases: (1) registration, (2) authentication, and (3) data storage. In this layer, message exchanges are handled between three entities: IoT devices, blockchain, and the cloud server (CS).

#### 4.2.1. Phase 1: IoT Device Registration

In this phase, all the IoT devices register with the cloud for secure data storage. Each IoT device follows the procedure below:

1. IoT device $d_{n}$ registers with ${ID}_{n}$, and the device type $d_{t}$*t*, computes the secret key ${sk}_{n}=h({ID}_{n},d_{t})$. The determined ${sk}_{n}$ and ${ID}_{n}$ are sent to the cloud via a secure channel.
2. Once the cloud receives $\left\{{ID}_{n},{sk}_{n},R\right\}$, it requests the location information of $d_{n}$. R denotes a random number. Then, $d_{n}$ computes its own location $L_{n(x,y)}$ and sends it back as $Pid=\{{ID}_{n}⊕L_{n(x,y)}\}$.
3. Upon receiving the location information, the cloud manages $\left\{{{ID}_{n},sk}_{n},Pid\right\}$.
4. Then, the PUF key $K_{F}$ is generated from a pair of public and private keys, ${Pk}_{n},{Pr}_{n}$ and ${Pk}_{n}$. These keys are asymmetric and in this work, ${Pk}_{n}$ is stored in the ledger required for authentication. The SRAM-PUF constructs a data structure as $D_{n}=\{{Pk}_{n},E_{K_{F}}\left(B\right),{ID}_{n},H\left({Pk}_{n}\right),A\}$. In this structure, $E_{K_{F}}\left(B\right)$ is the encrypted biometric of the device, A stands for attributes, and $H\left({Pk}_{n}\right)$ is the hash of the public key.
5. Then, $D_{n}$ is signed with ${Pr}_{n}$ and gives $S_{i}(D_{n})$. Thereby, this signed data structure is submitted.

Lastly, the IoT device is registered in the cloud, and its significant constraints are fed into the blockchain. Based on the transactions, the blockchain is maintained and authenticated accordingly.

#### 4.2.2. Phase 2: IoT Device Authentication

The registered devices are authenticated during data submission in this phase. The authentication process is conducted in the blockchain, which manages the records of the IoT devices. Transactions across IoT devices are recorded in a digital ledger. The steps for IoT device authentication are as follows:

1. The IoT device collects information and sends it to the cloud for storage. Initially, the device $d_{n}$ generates an authentication request with the timestamp $T_{i}$.
2. Upon receiving the authentication request ${Rq(d}_{n})$, the blockchain begins to authenticate $d_{n}$ by verifying the time interval $\left(T_{i}-T_{j}<∆T\right)$. Here, $T_{i}$ and $T_{j}$ are the current and estimated timestamps for a particular transmission, respectively. If the timestamp condition fails, the authentication request is dropped; otherwise, it proceeds. Next, the validity of ${Rq(d}_{n})=\left\{{ID}_{n},T_{i}\right\}$ is checked, determined, and sent to $d_{n}$.
3. After receiving A, $d_{n}$ checks the timestamp condition $\left(T_{b}-T_{j}<∆T\right)$. If this condition is satisfied, the process proceeds. Device $d_{n}$ checks the received $\left\{b_{id},T_{b},A\right\}$ and computes $S_{1}=h({ID}_{n}∥Pid∥R)$, then sends $\left\{{{ID}_{n},S}_{1},T_{i1}\right\}$.
4. The blockchain, upon receiving $\left\{{{ID}_{n},A}_{1},T_{i1}\right\}$, first verifies the timestamp and then extracts $S_{1}$ it for authenticating the location information. If the location information is valid, a message for $D_{n}$ is sent, including $\left\{b_{id},{ID}_{n}\right\}$.
5. The device $d_{n}$ Upon receiving the valid authenticated factors of the secret key and location, the final authentication of the IoT device with PUF is performed. $d_{n}$ reconstructs the noisy PUF and a fresh approach, devices, and ${Pr}_{n}$ keys. The corresponding encrypted $E_{K_{F}}\left(B\right)$ is then extracted from the ledger. After computation, $d_{n}$ sends $h({ID}_{n},D_{n},freshB)$ via a secure channel.
6. Upon receiving the last security factor from the device, authentication is performed by validating ${(D}_{n},freshB)$ with respect to the blockchain ledger. If the security factor matches, the device is authenticated and allowed to store data in the cloud; otherwise, access is denied.

#### 4.2.3. Phase 3: Data Storage

In this phase, the lightweight elliptic curve Diffie–Hellman algorithm is presented to secure data storage from IoT devices. All authenticated devices are guaranteed permission to access cloud storage. Given the resource constraints of IoT devices, a lightweight algorithm is employed to ensure secure data storage. Initially, a key agreement is established between two entities, and then the data are encrypted by the IoT device for storage in the cloud. This algorithm operates over an insecure channel.

Let a and b be the parameters of the elliptic curve, with a base point selected from $G=\{x_{1},y_{1}\}$ that ranges in the order of On, where On∗G=0. Select On as the smallest positive integer and $E_{q}(a,b)$ is determined from the elliptic curve parameters.

Algorithm 1, depicted below, defines the working procedure of the elliptic curve Diffie–Hellman. The authenticated IoT device exchanges security parameters ${SP}_{d}$ and ${SP}_{s}$ of the device and cloud server respectively. The validity ${SP}_{d}$ is verified, and then it is permitted to store the encrypted data into the cloud. In this first level, the IoT device is authenticated, and the data are encrypted before storage in the cloud. On the other hand, IoT users access securely stored data from IoT devices. Due to the storage of sensitive information, users are also authenticated in the second layer. Even though the data are securely stored in an encrypted format, efficient authentication with multiple factors is required. Authenticating the IoT device using more than one significant factor ensures access by a legitimate IoT device. Further details on the authentication of IoT users are provided in the forthcoming section.
**Algorithm 1:** Elliptic Curve Diffie–Hellman1. Begin 2. Initialize authenticated IoT devices $d_{n}$ 3. $d_{1}$ computes $E_{q}(a,b)$ and determine ${SP}_{d}$ 4. $d_{1}$ sends ${SP}_{d}$ to CS 5. CS computes $E_{q}(a,b)$ and determine ${SP}_{s}$ 6. CS sends ${SP}_{s}$ to $d_{1}$ 7. If (${SP}_{d}$) is valid           {                    Storage access granted.                    goto step 9           else                    Re-establish key exchange.                  } end if 8. Encrypt the data and transmit via secure channel 9. End

### 4.3. Second Layer Authentication

In this second layer, IoT users are authenticated by considering multiple unique factors that represent a particular individual’s ability to access the cloud. The major factors considered for user authentication include identity, password, signature, and biometric information. This work uses two biometrics, iris and finger vein patterns, which are crucial in defining the originality of the users. Most previous works have considered fingerprints as a biometric, having become increasingly vulnerable to hacking. This work employs two biometrics that are difficult for attackers to compromise.

The second level of authentication comprises three phases: registration, authentication, and data access. Only registered IoT users are allowed to log into the system after passing the authentication process. The three consecutive phases are described as follows.

#### 4.3.1. Phase 1: IoT User Registration

In this phase, the user is registered with their security credentials. As discussed above, multiple factors are individually stored in the cloud and form a blockchain structure. IoT user registration is executed based on the steps listed below:

1. Let $U_{N}$ be the user whose identity and password are chosen by oneself as ${ID}_{N}$ and ${PW}_{N}$, respectively. These are the two basic constraints that are initiated for registration. $U_{N}$ computes $F_{1}=h({ID}_{N},{PW}_{N})$ and sends it to the cloud via a secure channel.
2. Upon receiving $F_{1}$, the cloud server (CS) creates a block and then collects other factors from $U_{N}$.
3. Then, $U_{N}$ submits $\left\{F_{2}{,F}_{3},F_{4}\right\}$ by computing $F_{2}$ from ECDSA, $F_{3}$ from iris, and $F_{4}$ from finger vein. $U_{N}$ computes $h\{F_{2}{,F}_{3},F_{4}\}$ and sends it to CS.
4. After receiving all four factors from $U_{N}$, they are uploaded into the blockchain, and further, the IoT user transactions are recorded and authenticated by the blockchain.
5. Lastly, a successfully registered message is delivered to the IoT user, and further authentication is performed in the next phase.

#### 4.3.2. Phase 2: IoT User Authentication

In this phase, the registered users are authenticated if they need to access data from the cloud. To ensure access for only legitimate users, four levels of authentication factors are presented. Each level authenticates with its corresponding factor and then moves on to the next. User access is permitted only when all four factors are authenticated by the blockchain. Like IoT device authentication, IoT users are also authenticated in the blockchain.

##### Level 1

The first level of authentication considers user identity and password. These two are the most common security credentials used in many research works and even in real-time applications. However, submitting the password is vulnerable to password-guessing attacks and recent keystrokes. To mitigate such vulnerability in passwords, a novel matrix-based password enrolment is proposed in level 1, which the user needs to enter. Here, one is not required to enter their original password but select the appropriate alphabet/symbol/integer.

A matrix-based password entry panel appears for password submission in this proposed first-level authentication. To strengthen level 1, a 4 × 4 matrix is displayed, as using a 3 × 3 matrix may be familiar to hackers, making a larger matrix preferable. The logic of this panel is to pick individual characters of the password by choosing corner values from the row and column.

**Figure 3:**
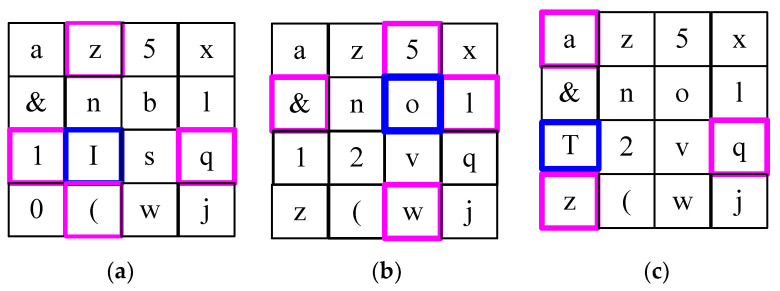
Matrix-based password panel: (**a**) intersection point ‘I’; (**b**) intersection point ‘o’; (**c**) intersection point ‘T’.

The defined matrix is composed of 16 individual characters, including the characters of your password. A sample matrix-based password entry panel is shown in Figure 3. Assume the password of $U_{N}$ is ‘IoT’. Refer to example Figure 3a, where the pink-coloured matrix boxes represent the cornered characters, whose intersection is predicted as ‘I’, i.e., the first character of $U_{N}$. Similarly, the other two characters are selected as the password from the matrix shown in Figure 3b, where the predicted point is ‘o’. Upon completion of each password entry, the matrix shuffles based on the password characters. Refer to example Figure 3c, where the pink-coloured matrix boxes represent the cornered characters, whose intersection is predicted as ‘T’. It is also shuffled for a particular user during their subsequent logins.

In this way, level 1 authentication is carried out. If $F_{1}=\{{ID}_{N},{PW}_{N}\}$ is valid, then the user will proceed to $F_{2}$ authentication.

##### Level 2

The elliptic curve digital signature algorithm plays a key role in authenticating users at level 2. In this level, the signature and the challenge–response (C-R) pair are enrolled along with the signature. According to the feedback from cloud validation using fuzzy logic, the C-R pair is strengthened. This level 2 authentication is carried out by the following steps:

Step 1: Let ${Pr}_{N}$ be the private key of user $U_{N}$, $B_{P}$ be the ECC base point, and O(N) be the order of the corresponding selected base point. Considering these three security constraints, $U_{N}$ generates a signature.
Step 2: Select a random integer r that ranges between them, then determine $k={\underline{x}}_{1}mod\;O(N)$. ${\underline{x}}_{1}$ is defined as an integer, and if the estimate k=0, then it begins with the selection of a different random number.
Step 3: Compute $r^{-1}mod\;O(N)$ and then calculate $h{\left(CR\right)}_{1}$, and then transform this into an integer e.
Step 4: Determine $s=r^{-1}\left(e+{Pr}_{N}.k\right)mod\;O(N)$. In the case where the computed signature s is 00, then go to step 1. The generated signature (k,s) is sent to the blockchain for authentication.
Step 5: If the signature from user $U_{N}$ and the previously registered signature is validated and is original, the user authentication is taken to the next level, or else it is dropped.

As per this level, the C-R pair is altered according to the validation feedback that is determined by fuzzy logic. If any one factor’s authentication fails, that user is assumed to be a suspect. Hence, to make stronger authentication for future access, the C-R pair is strengthened.

##### Level 3

In level 3, the authentication is handled with $F_{3}$, i.e., a biometric. At this level, the iris is considered as a biometric, and its pixel values are converted into binary values. An iris is captured as an image, and then the iris is represented as pixel values in matrix form. In this work, the images are transformed into binary values as columns and rows for processing. For instance, the uploaded iris is transformed into M×m binary values. Hereby, M and m represent the number of rows (r) and the number of columns (c), respectively. A pair of r’s and c’s are chosen, and each pair is combined with an AND, then an OR operator. The third level of authentication is built with the following steps:

The user’s text is mostly clear, but they may want to make sure that the terms and symbols used are consistently defined and used throughout the document. Moreover, it is important to ensure that the user’s audience is familiar or has been introduced to the terms and concepts they are discussing. It might be helpful to provide more detailed explanations or examples of the operations and transformations mentioned in order to aid comprehension.

Step 1: Assume the binary value is formed as a 8×8 binary value. Let the $U_{N}$ binary matrix be

$$U_{N}\left(M\times m\right)=\left[\begin{array}{cccccccc} 1 & 0 & 1 & 1 & 1 & 0 & 0 & 1\\ 1 & 0 & 1 & 0 & 1 & 0 & 1 & 0\\ 0 & 1 & 0 & 1 & 0 & 1 & 0 & 1\\ 0 & 0 & 0 & 1 & 0 & 1 & 1 & 0\\ 1 & 1 & 1 & 1 & 1 & 0 & 0 & 1\\ 0 & 0 & 1 & 1 & 0 & 1 & 0 & 1\\ 1 & 1 & 0 & 0 & 1 & 1 & 1 & 0\\ 0 & 1 & 1 & 0 & 0 & 1 & 0 & 1 \end{array}\right]$$

In this matrix, there are eight rows and eight columns, where a particular pair of row columns is extracted. According to the iris, the binary values are varied.

Step 2: Let the selected pair of rows and columns be ${(r}_{1},c_{1})$ and ${(r}_{3},c_{4})$. The corresponding binary values in $r_{1}$ and $c_{1}$, are applied with the AND operator; similarly, on the other hand, $r_{3}$ and $c_{4}$ are also processed. The processing is expressed as

$$\begin{array}{c} b_{1}=r_{1}\;\&\&\;c_{1}\\ \;=[10111001]\;\&\&\;[11001010] \end{array}$$

$$\begin{array}{c} b_{2}=r_{3}\;\&\&\;c_{4}\\ \;\;=[01010101]\;\&\&\;[10111100] \end{array}$$

Step 3: After estimation of individual binary values as from $b_{1}$ and $b_{2}$, these values are correlated using the XOR operator for determining binary key $b_{KY}$. Lastly, in this level, $b_{KY}$ is computed as follows:

$$b_{KY}=(b_{1})⨁(b_{2})$$

Step 4: The estimated 8 bit $b_{KY}$ is hashed and sent to the blockchain for authentication. If the $b_{KY}$ is valid, then the IoT user is authenticated with the final level.
Step 5: As per this level, the computation is simpler with operators and complex for the intermediate party to determine this key. If the security is leaked, then the new binary key can be generated with the permitted access from the cloud. Later, the changed key is updated in the blockchain.

##### Level 4

The fourth level of authentication considers finger vein patterns, which are unique to everyone. Finger vein biometrics are emerging as reliable tools for authentication, ensuring enhanced security and accuracy alongside long-term stability. The uniqueness of finger vein patterns, which remain distinct even among identical twins, guarantees their inability to be duplicated. This is a significant advancement in fingerprint biometrics, which has been undermined by security concerns and issues of authentication accuracy due to skin damage.

Unlike fingerprints, finger vein patterns are not vulnerable to external injuries, consistently yielding exact authentication results. In the authentication process, if an IoT user passes the first three levels, the finger vein pattern is then matched by calculating the pixel distance difference. Successful authentication at this level grants the user access to the cloud to retrieve data. The Euclidean distance measure is employed to assess the similarity between the input and stored finger vein patterns, ensuring a robust and secure authentication process.

$$d\left(x,y\right)=\sqrt{\sum_{i=1}^{n}{\left(x_{i}-y_{i}\right)}^{2}}$$

From the above-mentioned distance formula, the similarity between finger veins is predicted. The distance between *x* and *y* vectors is determined from two-pixel points within an *n*-dimensional space. In this manner, the Level 4 authentication involving finger veins is handled. If authentication is successful across all four levels, the user is granted permission to access data from the cloud. It is important to note that all factors are hashed before being sent to the blockchain.

#### 4.3.3. Phase 3: Access Cloud Data

IoT users are permitted to access the cloud only when all four levels are accurately matched, and they are verified as legitimate users. If an IoT user fails in any one aspect of authentication, they are allowed to restart the authentication process from the initial level. Fuzzy logic is utilized to validate the authentication and identify any unauthorized or suspicious users attempting to access a particular user’s data. After this validation, feedback is relayed to the second level of authentication. This level incorporates signature generation along with the challenge–response pair to preclude illegitimate user access. Based on fuzzy rules, all membership rules are constructed with the authentication results of the four factors.

**Figure 4:**
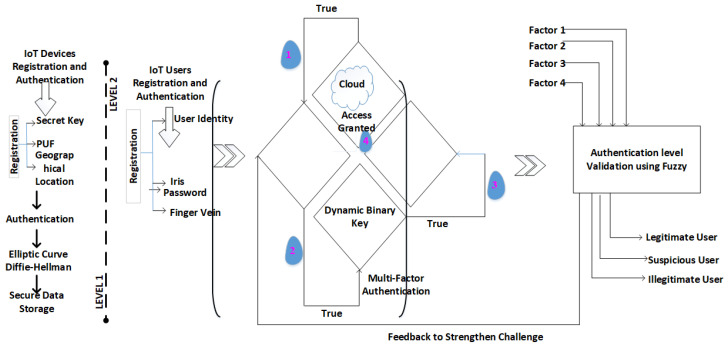
Workflow of proposed 2L-MFA.

Fuzzy logic is used in the validation process to categorize users as legitimate, suspected, or illegitimate. A user authenticated on all factors is deemed legitimate, failure on all counts marks a user as illegitimate, and failure on any one factor labels a user as suspected. This validation process aids in enhancing security levels by preventing unauthorized users from gaining access to the cloud. Figure 4 provides a comprehensive overview of the proposed 2L-MFA system, highlighting the use of blockchain for authentication.

Algorithm 2, depicted below, pseudo code addresses the entire process of 2L-MFA proposed for authenticating both IoT devices and IoT users via blockchain. Blockchain plays a major role in authenticating entities using appropriate factors. The involvement of multiple security factors ensures security in the designed IoT cloud environment.
**Algorithm 2:** Pseudo Code: 2L-MFA1. Start L1 Authentication // Begin Level 1 authentication 2. Initialize d_n    // IoT devices to be authenticated 3. 〖Rq(d〗_n)→Blockchain    If (〖 T〗_i-T_j<∆T)     // Timestamp verification      {        If 〖(ID〗_n=Valid)  // Authenticating first factor ID          {          Check next factor // Verify second factor        else          Drop request    // Invalid factor          }     else        Drop request       }     end if 4. If 〖(T〗_b-T_j<∆T)     // Timestamp verification     {       If (〖 S〗_1= True) //Authenticating second factor secret key         {        Check next factor  // Verify third factor       else        Drop request    // Failed authentication         }   else     Drop request      }  end if 5. If (L_(n(x,y))=True)    // Authenticating third factor location    {     Check next factor   else     Drop request     }   end if 6. If (E_(K_F ) (B)=True)  // Authenticating fourth factor PUF   {     Store Encrypted Data // lightweight ECC Diffie-Hellman   else     Drop request     }   end if 7. Stop L1 Authentication  // Finish level 1 IoT device authentication 8. Start L2 Authentication     // Begin level 2 authentication 9. Initialize U_N          // IoT users to be authenticated 10. If 〖(ID〗_(N,) 〖PW〗_N= valid)  // Authenticating First factor ID, password     {       go to next factor   // Verify the second factor      else       drop request       }   end if 11. If (k,s)= valid   // Authenticating Second factor signature and CR      {        go to next factor    // Verify the third factor    else        drop request      }   end if 12. If (b_KY=True)       // Authenticating third factor binary key from iris      {        goto next factor  // verify fourth factor      else        drop factor       }   end if 13. If (FV=valid)       // Authenticating fourth factor finger vein        {         Access granted       else         Access denied        }   end if 14. Stop L2 Authentication  // Finish level 2 IoT device authentication

## 5. Result and Discussion

**Figure 5:**
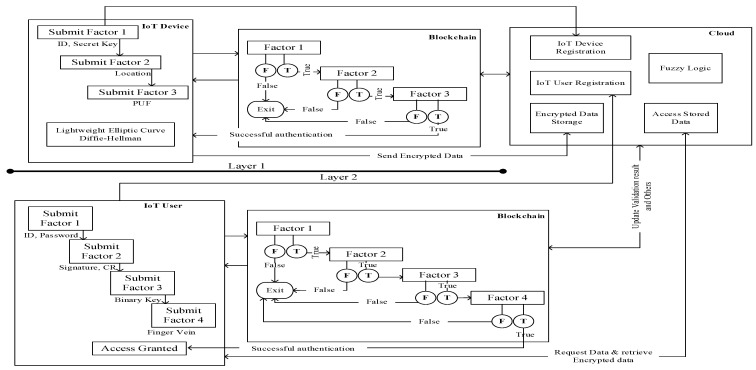
Pipeline structure of the proposed 2L-MFA.

This section deploys the research outcomes of the proposed system. Results attained for the 2L-MFA are depicted in this section, which is categorized into implementation setup, performance analysis, and security analysis, where Figure 5 represents the pipeline structure of the proposed 2L-MFA.

In Table 2, the major parameters that are considered for developing 2L-MFA are presented. The specifications are not limited to time; they also include all other common constraints. The numbers of users are able to be larger, and this system is adaptable for a huge participation of users. However, when the number of users is increased, authentication time is not accurately reflected due to the presence of blockchain technology. ECC key size with 256 bits enables the attainment of 128 bits of security level with long-term protection.

### 5.1. Performance Analysis and Comparison

This section illustrates the performance of the proposed 2L-MFA with respect to previous IoT-based authentication systems. This paper focuses mainly on authentication in IoT using blockchain technology. Our comparison is held on significant constraints of security and time. According to the proposed 2L-MFA, the IoT cloud aims to ensure appropriate authentication with multiple factors that disable the involvement of illegitimate entities in the system. IoT is widely used in several applications such as healthcare, smart city, and others. Hereby, most of the applications share sensitive information that is essential to be covered with secure access.

For authentication, the constraints considered are time and authentication success rate. As for the proposed 2L-MFA, the time is individually evaluated for each phase. Most of the research works have concentrated security either on IoT devices or IoT users, and from our knowledge, this is the first work to focus authentication on both IoT devices and IoT users.

#### 5.1.1. Time

Time plays a key role in provisioning security, i.e., authentication. The three main phases involved in this comparison are the registration phase, authentication phase, and login phase. Once the users are authenticated with all the factors, then they are immediately logged into the system to access data. The time for all these three phases is estimated, and the results are compared.

In previous work [26], an authentication scheme in IoT cloud was established by considering identity, password, and random number as its security factors. They are collectively maintained into smart cards, which are a special entity used for authentication. Smart cards are extensively used in many state-of-the-art research works for promising authentication. However, it is required that they are carried, and if the smart card is lost or stolen, then it can be used by an illegitimate user, and even the password of that user can be changed. The revocation of this smart card is essential, and using common security credential identity will lead to poor security. To overcome the problem of smart cards, the proposed 2L-MFA IoT cloud used SRAM-PUF, which is built in IoT devices, and it is unclonable. Along with this, other unique security credentials are considered for IoT devices.

**Figure 6:**
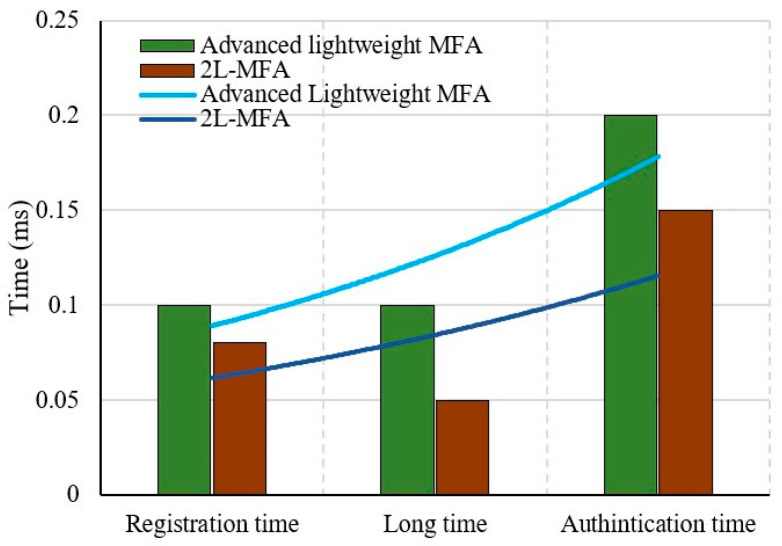
Comparison of registration time, authentication time, and login time.

Figure 6 demonstrates the comparative results of registration, authentication, and login time. From this comparison, the proposed 2L-MFA in blockchain has achieved lesser time consumption than the previous work. Up to 25% of registration time, 50% of the login time, and 25% of the authentication time are minimized. The login time is comparatively lesser than the authentication time since after completion of authentication, access to the cloud is granted without any delay.

#### 5.1.2. Authentication Success Rate

The authentication success rate depends on the number of users who request access to the cloud. In some worst cases, there is a possibility of misauthenticating legitimate users as illegitimate. Based on the potentiality of the security factor, the authentication access rate is enriched.

**Figure 7:**
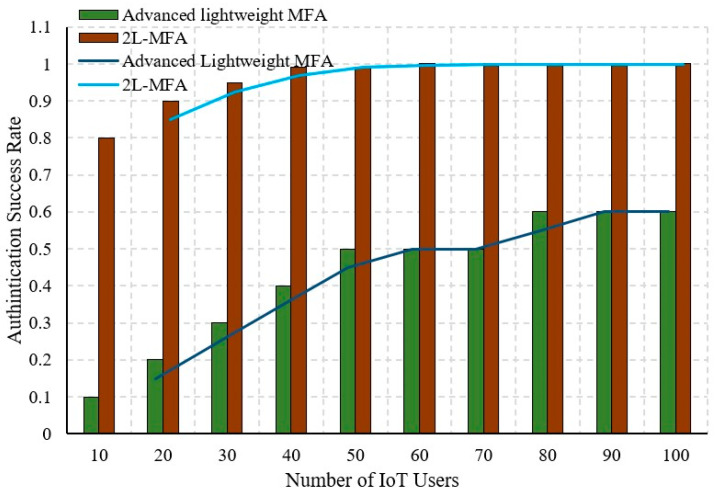
Comparison of authentication success rate.

The authentication success rate is measured and compared in Figure 7, which depicts a higher success rate than previous work. The higher success rate shows that all legitimate users are authenticated correctly, and illegitimate users are ignored. The involvement of blockchain in this proposed work prevents the authorization of illegitimate users. The main advantage is the consideration of security factors that are unique to identify individuals. Even the longer time for authentication also leads to a poor authentication access rate. In the proposed 2L-MFA, all the users are correctly authenticated with respect to the increase in the number of requesting users. Minimized computation in authentication also reflects an increase in authentication access rate.

#### 5.1.3. Security Constraints Analysis

In this section, the significance of the proposed authentication is highlighted from previous research work. Recently, most of the IoT cloud environments have been deployed with security due to the challenges faced in this environment. For authentication, certain factors are considered to check the originality of a particular. However, security credentials play a key role, and they need to be chosen appropriately. The proposed 2L-MFA is a promising solution for authentication, which brings legitimate user access into the system. The deployment of blockchain and SRAM-PUF into this proposed system is equipped to attain a higher level of security. Due to the increased number of threats and attacks in IoT, it is essential to resolve this challenging aspect with a multi-factor authentication scheme.

Table 3 depicts a comparison of five security factors, namely, identity, password, smart card, biometrics, and PUF. Knowing the disadvantage of smart cards, 2L-MFA have ignored their use and equipped IoT devices with PUF, which is focused on limited previous works. The built SRAM-PUF is unclonable and provides authentication with a unique biometric. Identity and password are the two major security factors that are easily hackable and even guessed. Biometrics are a satisfactory solution for authentication, but previous works have used fingerprints as a biometric, which is easily hacked in the current era. The complete authentication is handled on blockchain, and lightweight computations ensure the awareness of energy and resources of IoT devices.

Blockchain in our system is supported to cope with lightweight IoT devices by deploying the concept of proof of authentication (PoAh) [39]. This PoAh verifies the entities with respect to the factors by lightweight block verification. The advantages of PoAh include minimizing energy consumption, reducing computation, and reducing latency. The registered devices and users are enabled to maintain the distributed ledger as in blockchain, and the corresponding transactions must be updated. The two-step authentication presented in PoAh is block authentication and block validation. The potential of PoAh in the proposed 2L-MFA builds the assurance of end-to-end security in the designed infrastructure.

Table 4 depicts the following vulnerabilities: V1—mutual authentication, V2—malicious user attack, V3—forward secrecy, V4—password guessing attack, and V5—smart card revocation for comparing the performance of the proposed in accordance with previous authentication systems. As per the considered security factors, the authentication is modelled. In some works, smart cards are considered a factor that failed to design an appropriate revocation procedure. In the proposed 2L-MFA, the smart card is not considered, and the smart and revocation process is not present (NP); apart from this, the proposed work is able to overwhelm any attack.

### 5.2. Security Analysis

In this section, the secure authentication provisioned in 2L-MFA is highlighted in terms of forward secrecy, integrity, mutual authentication, and confidentiality. The role of the proposed 2L-MFA is to ensure authentication for legitimate IoT entities and deny illegitimate IoT entities’ participation in the system. In this security analysis, the potentialities of the proposed 2L-MFA authentication and their corresponding factors are studied.

#### 5.2.1. Forwarding Security

The proposed 2L-MFA provides forward secrecy for both IoT devices and IoT users since blockchain is intermediate, wherein authentication is handled before reaching the cloud. During registration, either a secure channel is used, or the security constraints are hashed, so that registration and authentication in 2L-MFA assist forward secrecy.

#### 5.2.2. Resistance to Man-in-Middle Attack and Impersonation Attack

In level 1, the biometrics of IoT devices are encrypted before being stored in the ledger, which ensures overwhelming the man-in-middle attacks on communication. The transmission of security parameters between server and device in terms of hashed values results in the avoidance of man-in-middle attacks.

In level 2, the users are validated with unique factors, and the biometric iris and finger vein in particular, are not able to be duplicated. On the other hand, the matrix-based password entry method ensures that a malicious person cannot guess the password. Hereby, the use of significant security factors is associated with overwhelming man-in-middle attacks.

Next, in level 1, the authenticated device and server exchange key are used before data storage. The exchange of a key followed by encryption using elliptic curve Diffie–Hellman algorithms enables security against impersonation attacks. On the other hand, in layer 2, the authentications are validated and strengthen the second factor through a breakthrough impersonation attack.

#### 5.2.3. Integrity

Integrity is nothing but the trustworthiness of the data, which means that the unauthorized entity can never alter the data. In 2L-MFA, integrity is achieved since a higher level of authentication is equipped with strong and unique factors that avoid unauthorized participation in the system. In level 1, even though the device is authenticated, the data are encrypted using the lightweight elliptic curve Diffie–-Hellman before storing it in the cloud. So, in level 2, the authenticated IoT user will receive encrypted data that needs to be decrypted to extract the original data. Hence, integrity is assured in this proposed 2L-MFA on the IoT cloud.

#### 5.2.4. Confidentiality

Confidentiality is a significant security requirement that defines the security of transferring data via wireless links. However, authentication of IoT devices and IoT users enables the participation of authorized entities, and it is essential to build the encryption of data in order to attain confidentiality. To satisfy confidentiality, the proposed 2L-MFA uses lightweight encryption, which encrypts the data, and on the other end, the data are decrypted. Hence, this security requirement is satisfied by 2L-MFA in IoT cloud.

## 6. Conclusions

In this paper, a novel 2L-MFA proposes an IoT cloud environment with the assistance of blockchain technology. Authentication is focused on both IoT devices and IoT users, i.e., on layer 1 and layer 2. Layer 1 authenticates identity, secret key, location, and PUF. Individual authentication of factors permits the IoT device to submit the data into the cloud after processing with a lightweight elliptic curve Diffie–Hellman algorithm. On the other hand, layer 2 is categorized into four sub-levels of authentication. The four factors of authentication in sequential levels are identity and password, signature, binary key, and finger vein matching. For password matching, a novel matrix-based password entry panel is presented to outperform attackers in accessing the cloud. The second level is based on the verification of the signature generated from ECDSA along with the challenge response. Then, the third level of authentication validates the binary key generated from the iris, and lastly, the finger vein biometric is matched using the distance between the pixels. Overall, the IoT user’s authentication is validated by fuzzy logic, and if any user is identified to be suspected, then their challenge response is strengthened during the next login access. Blockchain with PoAh is a promising solution for authentication in IoT infrastructure, and it has been deployed into the proposed work. Hence, the proposed 2L-MFA is enabled to address authentication in IoT with computation minimized and security increased system.

In future, this 2L-MFA is planned to be applied to applications and to define security factors based on their information sensitivity.

## Figures and Tables

**Table 1:** Notations used in this paper.

Symbol	Description
$d_{n}$	IoT device
$u_{N}$	IoT user
${ID}_{n}$	Device identity
${ID}_{N}$	User identity
${sk}_{n}$	Device secret key
$L_{n(x,y)}$	Location of IoT device
$\beta_{i}$	PUF key
${Pk}_{n}$	SRAM-PUF-based public key
${Pr}_{n}$	SRAM-PUF-based private key
$S_{i}$	Signature
$b_{id}$	Block identity
$T_{b}$	Block timestamp

**Table 2:** Parameters used in 2L-MFA.

Specification	Value
Number of IoT device samples	100–200
Number of IoT users	10–100
User password length	4–10 (alphabets/integer)
ECDSA length	192 bits
ECC key size	256 bits

**Table 3:** Comparison of authentication factors.

Reference	Identity	Password	Smart Card	Biometric	PUF
[9]	✓	✓	✓	✗	✗
[37]	✓	✓	✓	✓	✗
[38]	✓	✓	✓	✗	✗
[34]	✓	✗	✗	✗	✓
Proposed	✓	✓	✗	✓	✓

**Table 4:** Comparison of resistance to attacks.

Reference	V1	V2	V3	V4	V5
[9]	✓	✗	✗	✗	✗
[15]	✓	✓	✗	✓	NP
[16]	✓	✓	✓	✗	✗
[30]	✓	✓	✓	NP	NP
[32]	✓	✗	✓	✗	NP
[37]	✓	✗	✓	✗	✗
[38]	✓	✓	✓	✓	✗
[34]	✓	✓	✓	NP	NP
Proposed	✓	✓	✓	✓	NP

## Data Availability

Data are contained within the article.
